# Physicochemical Characterization, Stability and Cytotoxicity of a Blue Dye Obtained from Genipap Fruit (*Genipa americana* L.)

**DOI:** 10.17113/ftb.59.01.21.6809

**Published:** 2021-03

**Authors:** Camila Verly de Miranda Sabino, Bárbara Janaína Paula da Silva, Danielle Lima Bezerra de Menezes, Felipe Moura Araújo da Silva, Tatiane Pereira de-Souza, Hector Henrique Ferreira Koolen, Ádley Antonini Neves de Lima, Emerson Silva Lima

**Affiliations:** 1 Faculty of Pharmaceutical Sciences, Federal University of Amazonas, Av. Gen. Rodrigo Otavio, 6200, CEP 69077-000, Manaus, AM, Brazil; 2 Faculty of Pharmacy, Federal University of Rio Grande do Norte, Av. Sen. Salgado Filho, 3000, CEP 59078-970, Natal, RN, Brazil; 3 Institute of Exact Sciences, Federal University of Amazonas, Av. Gen. Rodrigo Otavio, 6200, CEP 69077-000, Manaus, AM, Brazil; 4 Metabolomics and Mass Spectrometry Research Group, University of Amazon State, Av. Carvalho Leal, 1777, CEP 69050-010, Manaus, AM, Brazil

**Keywords:** *Genipa americana*, genipap fruit, natural dye, geniposide

## Abstract

**Research background:**

The current commercial scenario indicates an increase in the demand for natural dyes. Compared to synthetic dyes, natural ones have the advantage of being sustainable, making them of great interest for the food and cosmetic industries. The development of new natural dyes is necessary, as well as the carrying out of complementary research regarding the existing ones.

**Experimental approach:**

The present study aims to characterize the physicochemical and biological characteristics of the dye obtained from dehydrated endocarp of the genipap (*Genipa americana*) fruit, as well as perform the relevant stability and cytotoxicity tests. The chemical characterization was performed by HPLC-MS/MS analyses. The stability studies were carried out by spectrophotometry and cytotoxicity assays using cell culture and fluorometric methods.

**Results and conclusions:**

After dehydration and milling of the fruit endocarp, water was added to the obtained powder (in the ratio 4:1) to extract the dye. Five compounds were elucidated using HPLC-MS/MS and confirmed the presence of the geniposide as its main compound. With the X-ray diffraction and electron microscopy analysis, we characterised the obtained powder as being amorphous and of porous structure with a variable size. The thermogravimetric analysis indicated a maximum loss of 61% mass after exposure to a temperature range from 240 to 760 °C. The obtained blue dye was stable in the absence of light, at room temperature and had neutral pH. In the cytotoxicity assay, (95.0±1.3) % of viable human fibroblasts were observed after exposure to this dye. The genipap fruit can be a viable alternative to produce a natural blue dye, since it is easy to obtain and has very low toxicity in food, pharmaceutical or cosmetic products.

**Novelty and scientific contribution:**

This study demonstrates for the first time the physicochemical and biological properties of a natural blue dye from *G. americana* fruit.

## INTRODUCTION

Natural dyes are distinguished by their biocompatibility, which makes them an alternative to the widely-marketed synthetic dyes (*1*, *2*). The growing commercial demand for natural substances, which have sustainable methods of production, has aroused interest in the search for new raw materials and/or improvement in the techniques used to extract these dyes (*2*, *3*).

The genipap (*Genipa americana* L.) is an angiosperma which belongs to the family Rubiaceae, of the order Gentianalis and is native to Central and South America (*4*). The fruit, when in its immature stage, is rich in a colorless iridoid called genipin. This substance acquires high reactive potential with amine groups when exposed to oxygen, resulting in the formation of an intense blue pigment (*3*). Genipin is easy to extract because it has good solubility in water and hydroalcoholic solutions, which has contributed to its historically widespread use by indigenous peoples as a dye of utensils and for body pigmentation (*5*, *6*).

Due to the increase in the demand for natural dyes, technological studies, such as those performed by Neri-Numa *et al.* (*3*), who evaluated the extraction of bioactive compounds from the genipap fruit, and those of Brauch *et al.* (*7*), who studied the obtaining and use of the dye from this fruit, are necessary in order to gain better knowledge of this raw material and its constituents, as well as defining it as a safe source. Therefore, the present study had the objective of physicochemically characterizing the dehydrated endocarp of the fruit, and performing *in vitro* cytotoxicity tests of the liquid dye, as well as evaluating its stability under changes of the storage conditions.

## MATERIALS AND METHODS

### Dye extraction

The immature fruits were collected at the site of the headquarter of the Brazilian Agricultural Research Corporation (EMBRAPA) located on the AM 10 Highway (km 28) (2°52'51.3'' S and 59°57'25.8'' W), state of Amazonas, Brazil, in the period from July to September 2016. The study was registered with the National System for the Management of Genetic Heritage and Associated Traditional Knowledge (*8*) under the number A3965C3. The fruits were washed in running water, followed by the removal and desiccation of the endocarp in an oven at 45 °C for 3 days. This material was processed in a knife mill until the powder was obtained, which was sieved by hand (*9*). The extraction variables, plant material mass fractions (5, 10 and 20%) and extractant (ethanol, water and ethanol/water at *φ*=0.5) were tested. The solutions were macerated for 7 days, and the absorbances at *λ*=590 nm were monitored using a spectrophotometer (T70 UV/Vis; PG Instruments, Da Nang, Vietnam) on days 0, 4 and 7 after extraction. A liquid blue dye was obtained, which was used for cytotoxicity and stability tests.

### HPLC-MS/MS analysis

All chemical analyses were performed on an HPLC-MS/MS system consisting of a liquid chromatography system (Accela, Thermo, Waltham, MA, USA) coupled to a triple quadrupole mass spectrometer TSQ Quantum Access equipped with an electrospray ionization (ESI) source) operating in positive mode. A Phenomenex Luna-C18 column (5.0 μm, 4.6 mm i.d., 150 mm) (Torrance, CA, USA) was used for chromatographic separation in the binary mobile phase. Solvent A was water and solvent B methanol (Tedia, Mexico City, Mexico). Gradient elution was performed at 35 °C from 0-15 min, 10-80% (*V/V*) B at a flow rate of 0.7 mL/min. The temperature of the autosampler was maintained at 25 °C and the injection volume was 15 μL. The ESI source parameters were previously optimized as follows: voltage of the ionization source 4.7 kV, main gas pressure 1.2·10^6^ Pa, auxiliary gas pressure 5·10^5^ Pa, scanning gas pressure 0 Pa, capillary temperature 200 °C, transfer capillary voltage 36 V, voltage of the lenses 120 V, microscan rate 4 ms, and maximum injection time 100 s. Argon (Praxair, Danbury, CT, USA) was used as collision gas, where collision energies ranged from 15 to 35%. We attempted to identify the compounds present in the fruit endocarp by manual interpretation of the MS/MS spectral data and comparison with previously published data (*10*).

### Fourier transform infrared spectroscopy

The resulting powder from the dehydrated endocarp was characterized by infrared spectroscopy using Prestige-21 IR equipment (Shimadzu Corporation, Kyoto, Japan) with attenuated total reflectance (FTIR-ATR) equipment. The analysis was performed in the region from 700 to 4000 cm^-1^, with 20 scans and resolution of 4 cm^-1^.

### X-ray diffraction

The powder diffraction profile resulting from the dehydrated endocarp was characterized in a Bruker D2 Phaser apparatus (Karlsruhe, Germany) using CuKα radiation (*λ*=1.54 Å) with a Ni filter and a 0.02° step, current of 10 mA, voltage of 30 kV, and the use of a Lynxeye detector.

### Scanning electron microscopy

Scanning electron microscopy was performed for morphological analysis of the obtained powder placed under double carbon tape and analyzed using a Hitachi tabletop microscope TM-3000 (Tokyo, Japan) with a minimum magnification of 200× and maximum of 1.0 k× at a voltage of 15 kV.

### Differential scanning calorimetry

Differential scanning calorimetry (DSC) measurements were performed on Q20 DSC cell (TA Instruments, Tokyo, Japan) using a hermetically sealed aluminium crucible. Approximately 4 mg of powder were used for all experiments under a dynamic nitrogen atmosphere (50 mL/min) at different heating rate (2.5, 5.0 and 10 °C/min) in the temperature range from 25 to 500 °C. The temperature and heat flow of the DSC instrument were calibrated with indium (melting point=157.5 °C and *ΔH*=26.7 J/g).

### Thermogravimetry/differential thermal analysis

Thermal analysis by thermogravimetry was performed on TGA-60H thermocouple (Shimadzu Corporation). The platinum crucibles were used with approx. 4 mg of the obtained powder under a dynamic atmosphere of N_2_ (50 mL/min) at a heating rate of 10 °C/min in the temperature range from 25 to 600 °C. Data were analyzed using TA-60WS software (*11*).

### Moisture content

*Genipa americana* dye powder (1 g) was weighed and analyzed in the moisture determination scale, model M5-Thermo (BEL Engineering, Milano, Italy) at a constant temperature of 100 °C.

### Stability study of the blue dye

The liquid dye, obtained by ethanol/water (*φ*=0.5) extraction, was filtered using vacuum. In order to suppress microbial growth, a 0.3% potassium sorbate (Sigma-Aldrich, Merck, St Louis, MO, USA) was used as the preservative. The stability test consisted of observing the behaviour of the dye with the changes of temperature (2-8, 22-27 and 42-47 °C) and pH (pH=4, 7 and 10), in addition to assessing the interference of light during the study. Absorbance was monitored with a T70 UV/Vis spectrophotometer (*λ*=590 nm; PG Instruments) on the first day (D0), after thirty (D30), sixty (D60) and ninety days (D90) of incubation under different experimental conditions.

### Evaluation of in vitro cytotoxicity

The cytotoxic activity of the liquid dye was evaluated on fibroblast (MRC-5) cell strains by Alamar Blue assay using resazurin sodium salt (Sigma-Aldrich, Merck) according to the method described by Ahmed *et al.* (*12*). Cells were obtained from the Rio de Janeiro Cell Bank, cultivated in Dulbecco's modified Eagle's medium (Sigma-Aldrich, Merck) at 37 °C, with 5% CO_2_ and plated on 0.5·10^4^ cells/well in 96-well microplates. To determine the IC_50_ values ​​(cytotoxicity index causing 50% cell death), the cells were treated with the dye at the concentrations of 100, 50, 25, 12 and 6.25 μg/mL. Dimethyl sulfoxide (Sigma-Aldrich, Merck) was used as a control. Subsequently, 10 μL of the 0.4% resazurin sodium salt solution was added to all wells of the treated plate for a period of 24 h. After 3 h of incubation, microplates were analyzed using the fluorescence mode (540 nm exchange filter and 585 nm emission filter) of a DTX800 microplate reader (Beckman and Coulter, Vienna, Austria).

### Statistical analysis

The data obtained in this study are presented as the mean value±standard deviation (S.D.) and analyzed statistically using the GraphPad Prism software v. 8.1.2 (*13*). Difference among the groups was compared using two-way analysis of variance (ANOVA) followed by Tukey’s test and considered significant at p≤0.05.

## RESULTS AND DISCUSSION

### Processing of Genipa americana fruit

The process was started with 20 kg of the whole green genipap fruit. Due to the higher deposition of genipin in the endocarp of the fruit (*14*), this part was used in the processing, finally obtaining 5.65 kg endocarp. In order to facilitate the packing and to control the microbial growth in this material, it was desiccated and then milled, which in turn resulted in 2.5 kg of raw powder. After sieving, it was classified as a coarse powder according to the Brazilian Pharmacopoeia (*9*), based on the retention of 50% of powder in a sieve with a mean diameter of 0.925 mm. According to Fonseca *et al.* (*15*), coarse powder is better for extraction of plant drugs, since very fine powders can compromise this process.

### Analysis of the extraction process

**Fig. 1:**
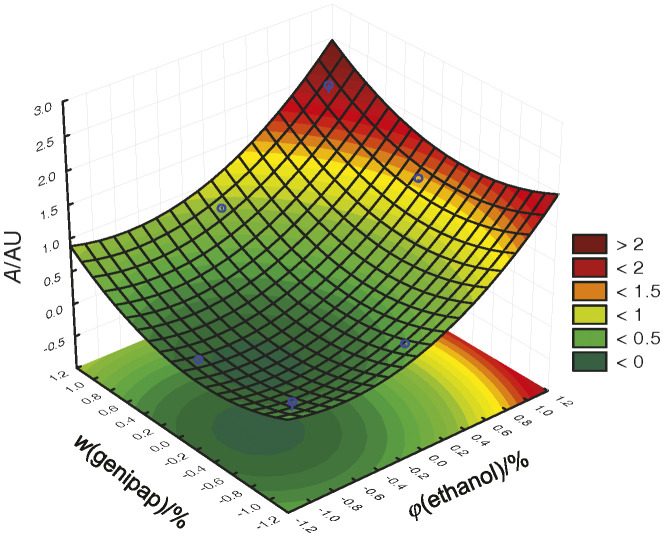
Factorial response surface study of the *Genipa americana* dye extraction parameters

Among the solvents tested in the extraction of the blue dye from the endocarp of the dehydrated fruit, water had a better extraction potential when the ratio of water to the dry endocarp was 4:1 (Fig. 1). Renhe *et al.* (*16*) performed the same procedure with water and ethanol as extractants. However, extraction with hexane was not possible, leading to the conclusion that the colour precursor compound is of polar origin. Water and ethanol are often recommended for the preparation of extracts due to their polarities (*17*). Water has a higher polarity than ethanol, and may justify better extraction of the dye (*18*). Genipin is present in the immature genipap fruit and is responsible for the formation of the blue colour through the reaction with amino acids in the presence of oxygen (*19*). The extraction performed by Neves *et al.* (*14*) resulted in bluish tones, which corroborate the findings of this study. Statistical analysis of the results showed that only the mass fraction of genipap powder was significant, confirming that a higher mass fraction of genipap powder and water as extracting liquid are the best parameters in order to obtain the blue pigment.

### Chemical characterization of the dye

**Fig. 2:**
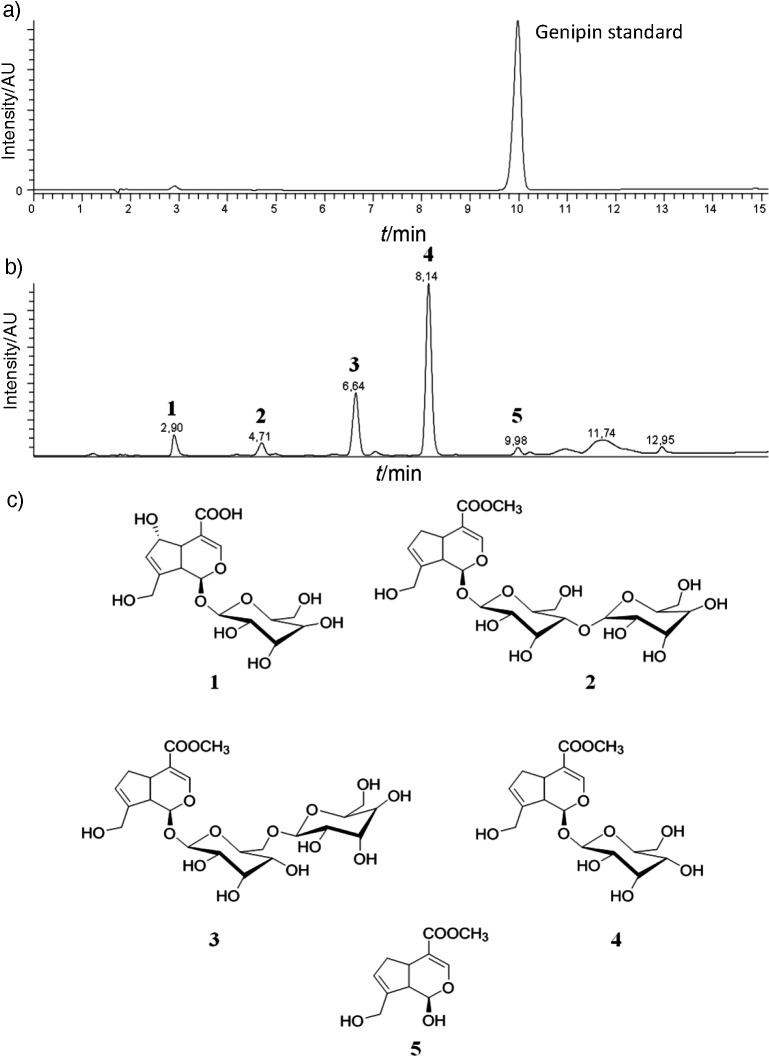
HPLC-MS/MS analysis of: a) *G. americana* fruit dye, b) genipine standard, and c) chemical structures of the compounds found mostly in the *G. americana* dye: 1=deacetyldaphylloside, 2=genameside C, 3=genameside D, 4=geniposide, and 5=genipin

The dye was chemically characterised firstly by injection of the genipin standard in the HPLC-MS/MS system, resulting in a chromatogram with a high peak at retention time of 9.98 min (Fig. 2a). Subsequently, the endocarp powder of the fruit was analysed, showing a higher retention peak at 8.14 min and the other at 9.98 min (Fig. 2b). Using structural elucidation, it was possible to identify five compounds present in the *G. americana* powder, which presented expressive peaks of retention in the HPLC chromatogram (Fig. 2b). After analyzing these results, we verified that the largest fraction of genipin present in the fruit was in its glycosylated form, the geniposide (number 4, Fig. 2c). According to Bentes *et al.* (*6*), genipin is an aglycone, resulting from the hydrolysis of the geniposide, through the enzyme β-glucosidase.

In order to identify the iridoid compounds present in the endocarp of the immature genipap fruit, Bentes and Mercadante (*20*) identified the prevalence of geniposide in relation to the others, followed by geniposide, gardenoside, shanzhiside and genipin. However, the study of the extraction of bioactive compounds by Náthia-Neves *et al.* (*21*) indicated a higher content of genipin in the endocarp and geniposide in the fruit mesocarp.

### Thermogravimetric analysis of the dye from G. americana

**Fig. 3:**
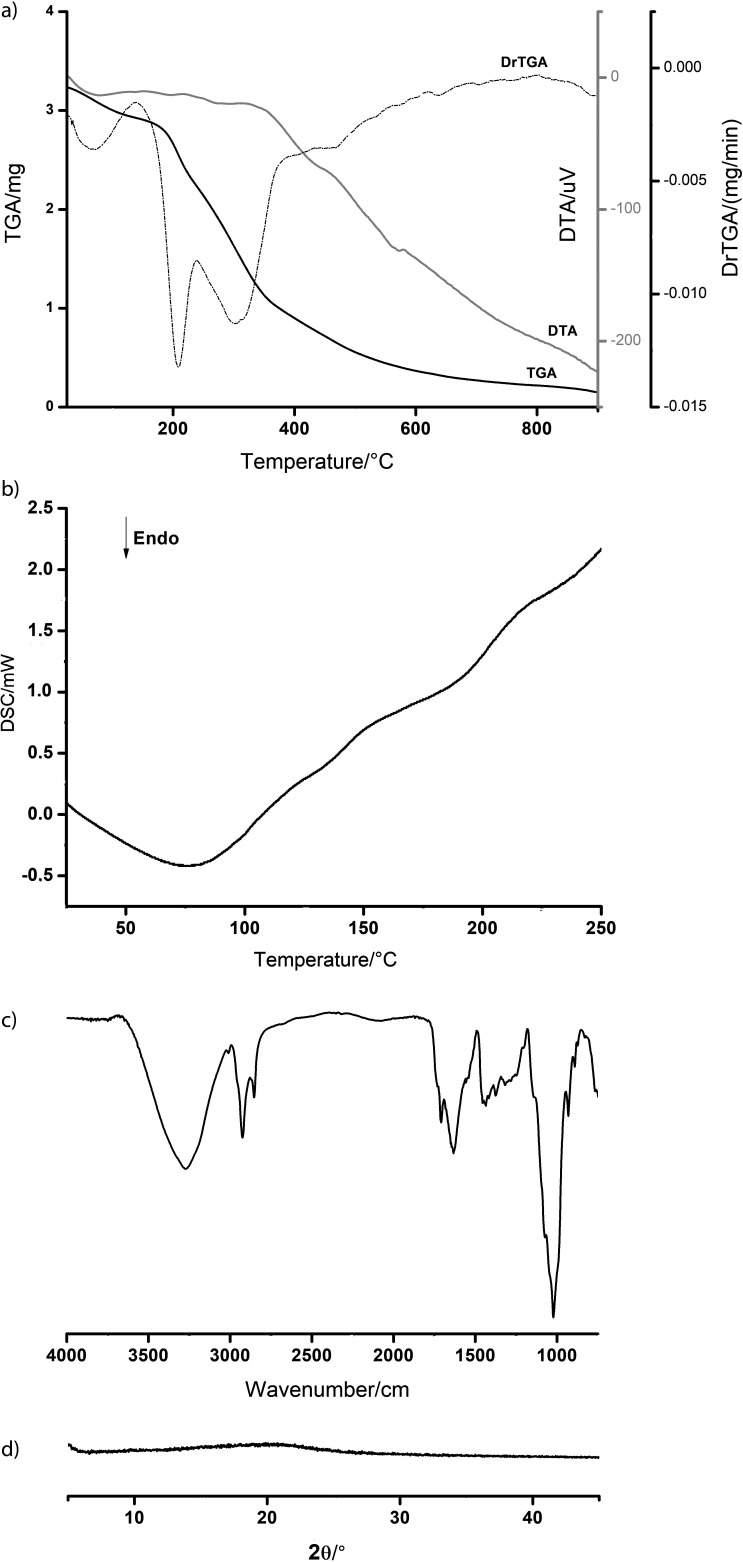
Thermal analysis by: a) thermogravimetry, b) differential scanning calorimetry, c) spectrum resulting from Fourier transform infrared analysis, and d) diffractogram of X-ray diffraction of *G. americana* dye. TGA=thermogravimetric analysis, DTA=differential thermal analysis, DrTGA=derivative thermogravimetry, DSC=differential scanning calorimetry

The thermogravimetric analysis of the dye from *G. americana* showed an initial endothermic event in the range of 27-95 °C, with a loss of mass equivalent to 6.5% related to the water loss previously observed by the dye moisture analysis (Fig. 3a). This phenomenon was also observed in the thermal characterization of the *Dicksonia sellowiana* extract performed by Malucelli *et al.* (*22*). Up to about 160 °C, the dye showed no significant mass variation, however, in the range between 163 and 220 °C, there was a mass loss equivalent to 19%, indicated by the peak in the differential thermogravimetric (DrTGA) curve at 211 °C when the mass varied more at a faster rate. From 330 °C onwards, a continuous mass loss of 40% was observed.

The DSC curve showed a marked endothermic event between 18 and 110 °C (*t*_onset_=21 °C, *t*_peak_=75 °C, *t*_endset_=102 °C, *ΔH* =52.39 J/g), which may be related to the loss of volatile constituents of the sample (Fig. 3b); in this case, the loss of water that was also observed in the thermogravimetric analysis. The decomposition process was progressive with increasing temperature, starting at approx. 133 °C. According to Fernandes *et al.* (*23*), degradation products of plant extracts can present different thermal behaviour due to factors such as loss of volatile components, sample heating rate and the presence of impurities that can directly interfere in the enthalpy of the obtained peak.

### Infrared and X-ray diffraction analysis of the dye from G. americana

FTIR analysis was performed to determine the functional groups present in the endocarp (Fig. 3c). The spectra obtained from the powder analysis showed bands around 3337 and 2946 cm^-1^, which correspond to the stretching of the -OH binding present in alcohols and polyphenols, whereas at 2834 cm^-1^ it refers to the elongation of the aliphatic CH, which can be attributed to the organic nature of the compounds present in the endocarp. At 1645 cm^-1^ band related to the C=O double bond of the aldehyde group -COOCH_3_ or the C=C conjugated, carboxyl group was observed. The band at 1449 cm^-1^ corresponds to deformation of the binding of the methoxy groups of genipin. The approximate region between 1112 and 1019 cm^-1^ corresponds to the sugar absorption bands related to the C-OH bond of the C-O-C group and the deformation of the hydroxyl CH_2_-OH group present in the geniposide. The results obtained are in agreement with those observed by Kumar *et al.* (*24*).

The X-ray diffraction analysis was performed to determine the degree of crystallinity of the obtained powder. Crystalline powders are characterized by a well-defined melting point and three-dimensional structure capable of refracting X-rays (*25*). Contrastingly, amorphous powders consist of randomly oriented molecules and diffract X-rays in all directions, resulting in the typical ’halo’ pattern, that is, the absence of crystalline reflections. The diffractogram of the powder of the endocarp is shown in Fig. 3d, in which a totally amorphous diffraction profile is observed, that is, there are no crystalline reflections in the diffractogram, confirming that it is a powder without any character or crystalline nature. This characteristic directly impacts the solubility of the powder, since amorphous particles are more easily solubilized in polar solvents, due to the random distribution of the molecules, favouring the wettability. The study by Gallo *et al.* (*26*) showed that the dried extract of *Rhamnus purshiana* also presented an amorphous profile.

### Scanning electron microscopy of the powdered dye from G. americana

**Fig. 4:**
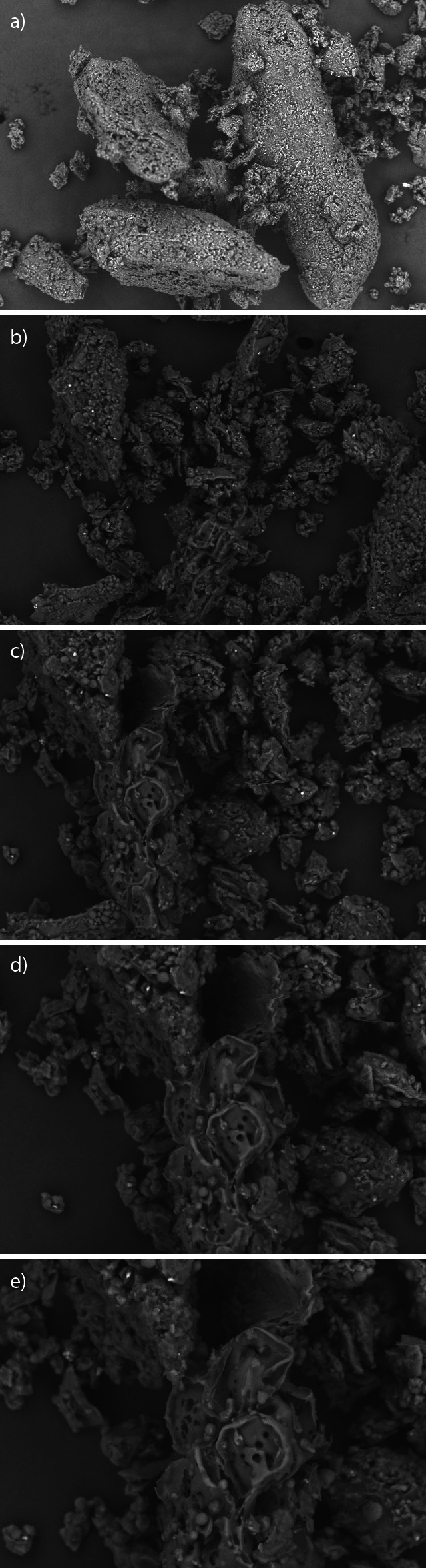
Micrographs of the *G. americana* fruit dye particles under different magnifications: a) 200×, b) 400×, c) 600×, d) 800× and e) 1.0 k×

Scanning electron microscopy is a qualitative analysis used to observe the surface texture of solids (*27*), in other words, to evaluate the morphology and particle size. The dye particles showed agglomerates of different sizes and shapes with an irregular porous surface, which is expected from the particles of plant extracts. These characteristics are common to the surface of amorphous compounds, corroborating what was observed *via* the X-ray diffraction, *i.e.* the absence of crystalline particles in the analyzed powder (Fig. 4). These characteristics may be directly related to the wettability properties of the powder (*28*).

### Stability of the dye from G. americana

**Fig. 5:**
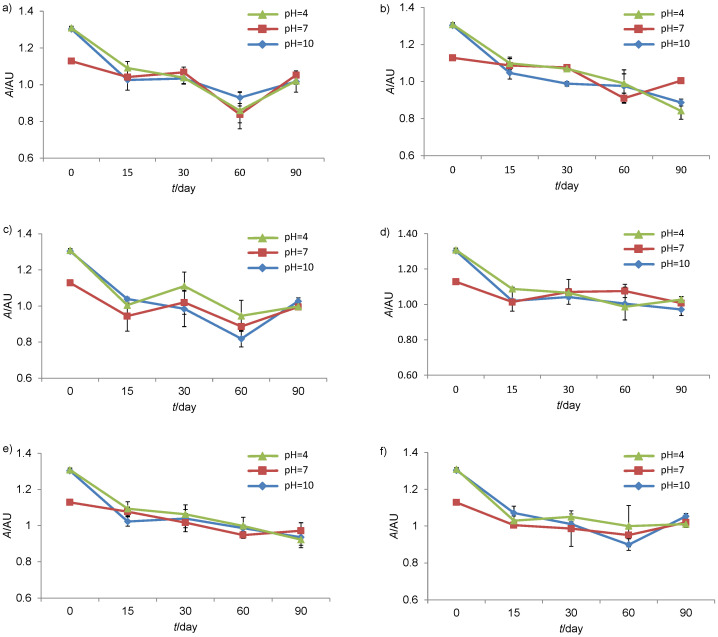
Stability of the *G. americana* fruit dye at: a) 20-25 °C in the dark, b) 42-47 °C in the dark, c) 2-8 °C in the dark, d) 20-25 °C exposed to white light, e) 42-47 °C exposed to white light, and f) 2-8 °C exposed to white light

In the stability test, the behaviour of the genipap aqueous dye was observed with temperature, pH and luminosity changes over 90 days (Fig. 5). Due to the aqueous nature of this dye, the addition of an antimicrobial preservative was necessary. The preservative potassium sorbate was chosen because its use in food and cosmetics is regulated by the Brazilian National Agency for Sanitary Surveillance (ANVISA) (*29*, *30*). The temperature variations caused visual changes in the product colour at the end of the analysis. At 45 °C, the solutions turned greenish, but the temperatures of 4 °C and ambient temperature did not change the initial colouration. These results corroborate those of Cho *et al.* (*31*), who observed that at room temperature the pigments remained stable; however, they lost 30% of their initial value after 140 h of exposure at 75 °C. Meanwhile, the study by Paik *et al.* (*32*) showed that, after 10 h of exposure to a temperature range of 60 to 90 °C, the pigments remained stable, which makes it necessary to monitor their stability for longer time.

The pH change did not interfere significantly with the stability of the dye (Fig. 5). However, when compared to each other, neutral pH kept the dye stable for longer time. Brauch *et al.* (*7*), who also evaluated the stability of the blue dye from *G. americana* fruit, noticed that the pH variation of the dye did not cause large changes throughout the process, demonstrating that this parameter does not significantly influence its stability. Cho *et al.* (*31*) analyzed the stability of the dye obtained from the reaction of genipin with amino acids within the range of pH=4-12 after 200 h of incubation at 55 °C and found that the change of pH value had no great influence, maintaining 80% of the initial absorbance.

While evaluating the exposure of the dye to the light, a decrease of its stability when compared to samples not exposed to luminosity was noticed. Paik *et al.* (*32*) evaluated the influence of light on the blue dye obtained by the interaction of genipin with phenylalanine, and this showed a loss of stability due to exposure to intense light. Likewise, in the study by Jespersen *et al.* (*33*), a degradation of the blue dye from *Gardenia* occurred when exposed to light.

### Evaluation of cytotoxicity of G. americana dye

**Fig. 6:**
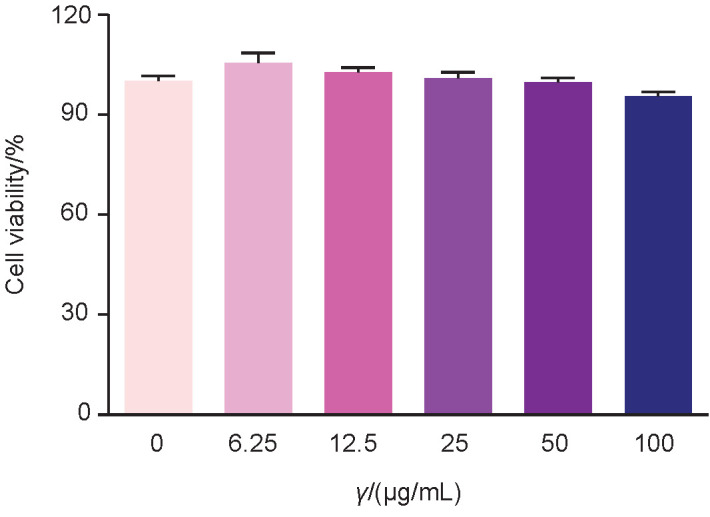
Cytotoxicity of the *G. americana* fruit dye in the MRC-5 fibroblast cells

MRC-5 human fibroblast was exposed to *G. americana* dye. In the cytotoxicity assay, the percentage of viable cells was (95.1±1.3) % at the concentration of 100 μg/mL of the dye, as shown in Fig. 6. Due to these results, the IC_50_ cannot be calculated, since even at the highest tested concentration, the death was not greater than 50%. This finding corroborates the belief that natural dyes are less harmful to health because they are biocompatible and therefore do not have a significant degree of toxicity, especially when compared to synthetic dyes (*33*). The cytotoxicity and neurotoxicity assays performed by Ab Kadir *et al.* (*34*) in the evaluation of the natural dyes from *Caulerpa lentillifera* and *Sargassum* sp. algae showed the lack of toxicity in these raw materials, once again showing the safety of the use of natural dyes.

## CONCLUSIONS

This study showed the feasibility of using genipap as a potential source for the production of a stable blue dye, which could easily be applied in the food, pharmaceutical or cosmetic industries. With the characterization of the endocarp of this fruit, we were able to identify two major compounds (geniposide and genameside D). This material has an amorphous, porous structure, which varied in size, and had a low moisture content. The extraction and dye stability analyses indicated the use of water as the best extraction solvent at a mass fraction of dry genipap endocarp of 20%, in addition to establishing room temperature, neutral pH and lack of luminosity as the ideal conditions for obtaining and maintaining the blue pigment stable. The biocompatibility of the produced dye was evidenced in the cytotoxicity assay, resulting in high cell viability after exposure to high mass fraction of dye. The work demonstrated that the dye from genipap fruit is a promising alternative to substitute synthetic dyes, since it has a sustainable production method, it is not harmful to the human organism, and can thus reduce cases of allergies that are widely attributed to the use of synthetic colourants. Subsequent studies should be performed in order to show details of the applications of this dye in medicines, food or cosmetics.
